# Social isolation in mental health: a conceptual and methodological review

**DOI:** 10.1007/s00127-017-1446-1

**Published:** 2017-10-28

**Authors:** Jingyi Wang, Brynmor Lloyd-Evans, Domenico Giacco, Rebecca Forsyth, Cynthia Nebo, Farhana Mann, Sonia Johnson

**Affiliations:** 1 Division of Psychiatry, University College London, Maple House, 149 Tottenham Court Road, London, W1T 7NF UK; 2 Unit for Social and Community Psychiatry, (WHO Collaborating Centre for Mental Health Services Development), Queen Mary University of London, London, UK; 3 Camden and Islington NHS Foundation Trust, St Pancras Hospital, 4 St Pancras Way, London, NW1 0PE UK

**Keywords:** Social isolation, Loneliness, Conceptual model, Measures, Mental health

## Abstract

**Purpose:**

Social isolation and related concepts have been discussed increasingly in the field of mental health. Despite this, there is a lack of conceptual clarity and consistency in the definition and operationalisation of these terms. This review aimed to provide a clear framework for social isolation and related concepts, and to identify well-established measures in the field of mental health for each conceptual domain discussed.

**Methods:**

We used an iterative strategy of expert consultation and literature searching. A multi-disciplinary group of senior academics was consulted both before and after the literature searching to identify relevant terms, conceptual papers, or recommended measures. Our conceptual framework was also validated through expert consultation. We searched the Web of Science database using terms suggested by experts and subsequently identified further relevant studies through review articles and by reading full texts and reference lists of included studies. A narrative synthesis was conducted.

**Results:**

We developed a model with five domains incorporating all the concepts relevant to social isolation in regular use in the mental health research literature. These five domains are: social network—quantity; social network—structure; social network—quality; appraisal of relationships—emotional; and appraisal of relationships—resources. We also identified well-developed measures suitable for assessing each of the five conceptual domains or covering multi-domains.

**Conclusions:**

Our review proposes a conceptual model to encompass and differentiate all terms relating to social isolation. Potential uses are in allowing researchers and intervention developers to identify precisely the intended outcomes of interventions, and to choose the most appropriate measures to use in mental health settings.

**Electronic supplementary material:**

The online version of this article (doi:10.1007/s00127-017-1446-1) contains supplementary material, which is available to authorized users.

## Introduction

There has been a growing realisation among policy makers and practitioners that social relations play an influential role in mental health and psychological wellbeing [1], and that service users themselves place high importance on them. Feelings of loneliness are greater and social network size is smaller among mental health service users than in the general population [2–5]. The previous studies have identified an association between loneliness and depression [6, 7], suicidal behaviour [8], personality disorders [9], and psychoses [10]. Among people with severe mental illness, social isolation has been linked to higher levels of delusions [11], lack of insight [12], and high hospital usage [13]. Conversely, people who report greater informal social support have been found more likely to recover from psychotic symptoms [14].

There is a lack of clarity around how social isolation, loneliness, and related concepts should be defined and measured [15, 16]. While social isolation has been linked to loneliness, they are not synonymous concepts [1, 17]. These, and related terms, including social networks, confiding relationships, and social support, have multiple, often overlapping, meanings. Due to this lack of clarity, researchers sometimes use these terms loosely and interchangeably [18]. In this review, we focus entirely on social relations as they are experienced at the individual level. A higher order sociological approach looks at how people relate to each other within a society. Concepts including ecological social capital, relating to the quality of social relationships within a community, social exclusion, relating to an enforced lack of participation in mainstream social, cultural, economic, and political activities and social inclusion, relating to individuals’ access to resources and participation in economic, political, and social activity, can be distinguished from concepts which focus on relationships at the individual level, such as social isolation.

Existing reviews have provided an overview of the current conceptual and methodological literature on social exclusion [19, 20] and social capital [21, 22]. In their 1988 review, House and colleagues identified the structures and processes through which social relationships influence health [23, 24]. Since then, a literature on social relationships and mental health has emerged, in which explicit reference to an overarching conceptual frame work is generally not made. Our goal in this paper is to examine the concepts in use in this literature, the extent to which they can be synthesised into a coherent framework, and the match between this conceptual framework and others applied to examining associations between aspects of health and social relationships. A recently published conceptual review investigated measures of loneliness, social isolation, and social relationships at the individual level, focusing on older adults and cardiovascular disease populations [18]. Our current review adds to this understanding, being the first to review the use and measurement of social isolation and related concepts in the field of mental health.

The aim of this review is to provide a comprehensive framework for social isolation and related concepts, and to identify examples of different measurement tools, highlighting the best established measures in the field of mental health. It can help future researchers to decide exactly what they want to measure and how to go about it.

## Method

### Overall approach

Conceptual and methodological reviews differ from systematic reviews of effects. The exact scope and procedures of conceptual reviews are established through the process of conducting the review. We followed Lilford and colleagues’ recommendations for conducting methodological reviews [25] and used an iterative and consultative process to achieve a clear understanding of social isolation and related concepts. This included: searching widely using a variety of databases and sources; making sure that the review is informed by expert advice, including social science, psychological and medical perspectives; allowing some overlap in the various stages of the review process, so that the final scope and findings of the review could be clarified in response to interim findings and feedback.

### Literature search

Our iterative search strategy involved: (1) expert consultation to identify relevant terms, conceptual papers, or recommended measures; (2) literature search, data extraction, and conceptual map development; (3) expert consultation to validate the conceptual framework. Detailed process is described below:

*Expert consultation* First, we consulted a multi-disciplinary group of London experts in social aspects of mental health to identify relevant terms for our literature search. Second, following the initial literature searching, we extracted data which informed the development of a draft conceptual map with several domains to fit all identified relevant concepts. Then, we consulted this same group and contacted 15 international experts identified through the initial literature searching, to present our draft conceptual map, seeking feedback and suggestions. These international experts have relevant subject expertise within and outside of the field of mental health.

*Literature search* Using terms suggested by the experts, we searched the Web of Science database on 23rd April 2015 for papers defining social isolation and related terms, or the methods of measurement for these concepts. Search terms for social isolation and related terms (social isolation OR loneliness OR social network* OR social support OR confiding OR confide OR social contact* OR social relation* OR social capital) were combined with terms for mental disorders [mental OR psychiatr* OR schizo* OR psychosis OR psychotic OR depress* OR mania* OR manic OR bipolar near/5 (disorder or disease or illness) OR anxiety]. Time limits for the initial search were restricted to 1st January 2013–23rd April 2015 as a high volume of articles was retrieved initially. Web of Science was selected as an inter-disciplinary database covering a wide range of subject areas. Reference lists of studies identified were hand-searched for other relevant studies, without time limit. Wherever a paper retrieved for full-text screening referred to another potentially relevant study, this too was retrieved and screened.

From the initial database search, studies proposing a definition or measure of a concept relating to social isolation and applying this concept or measure to the field of adult mental health were included. Studies of children under 16 and learning disability/organic disorder populations were excluded. Studies with no explicit definition of social isolation or related concepts, or those not using well-developed measures, e.g., single item, were excluded. Where concepts/measures used in a mental health context had originally been developed in other fields, the original source was retrieved and reviewed.

### Data extraction and synthesis

We extracted information on definitions of social isolation and related terms, and on approaches to its measurement, using an electronic data extraction form developed for this review. The initial screening was conducted by single review authors (JW, BLE, RF, CN, and FM), with regular meetings between review authors to address uncertainties about inclusion where necessary and check that a consistent approach to screening was applied.

A narrative approach was adopted to synthesise findings. This comprised three stages.

i. Review authors developed a set of conceptual domains covering all elements within identified existing conceptualisations of social isolation and related terms retrieved.
ii. The validity of this conceptual framework was then assessed referring to the existing literature. All included papers from the literature search were cross-referenced with the domains developed, to check whether our conceptual map was sufficiently comprehensive to include all relevant concepts and was not adding additional domains not covered in the literature. A record of the retrieved concepts which we reviewed and how we mapped them to the domains of our conceptual framework is provided in Supplementary File 1, Table 1–7.
iii. Measures of social isolation and related concepts identified by our literature search were reviewed and best examples of suitable measures for each proposed conceptual domains were identified. Measures with good established psychometric properties, demonstrated applicability, and wide use in mental health settings were prioritised. The initial selection of appropriate measures was undertaken by single review authors (JW, BLE, RF, CN, and FM); review authors met to agree the final selection based on above criteria.

Further consultation with experts was conducted to improve and validate the conceptual model and to identify any further relevant literature or concepts not included. We persisted in this process until no new concepts or measurement tools emerged.

## Results

Our electronic database search identified 5437 papers. After full-text screening of papers from electronic search and from reference lists and review articles, we included 277 papers discussing concepts relating to social isolation. We also retrieved 425 papers presenting measures of relevant concepts, including 191 original papers developing these measures. Of these, we have reported 16 in our review: those most widely used in the field of mental health, with the best established psychometric properties.

### Definitions and brief explanation of relevant concepts

In this section, a brief summary of the ways in which social isolation and related terms have been conceptualised is provided. A fuller description is provided in Electronic Supplementary Material 2. These concepts have been widely cited in mental health research, although not all of them originated in the field of mental health.

#### Social isolation

Models of social isolation include both objective social contact and subjective perceived adequacy of contact. Zavaleta et al. [26] defined social isolation as “inadequate quality and quantity of social relations with other people at the individual, group, community, and larger social environment levels where human interaction takes place”.

#### Loneliness

Loneliness is a painful subjective emotional state occurring when there is a discrepancy between desired and achieved patterns of social interaction [27, 28]. It is thus conceptualised as an entirely subjective state, not necessarily dependent on the quantity of someone’s social relations.

#### Social support

Two domains are usually distinguished and measured within the overarching concept of social support: functional social support, the functions fulfilled by social relations, and structural social support, the existence, quantity, and properties of social relations [29]. Many measures of social support assess three components, spanning both structural and functional domains: social network and social integration variables, received support, and perceived support [30, 31].

#### Social network

Social network refers to an individual’s connections among a group of people, the characteristics of which are used to interpret the social behaviour of people involved [32]. This includes objective, morphological characteristics such as network size, degree and density, and interactional characteristics including network intensity and directionality [33].

#### Social capital

Social capital is generally understood as a series of resources that individuals earn as a result of their membership in social networks, and the features of those networks that facilitate coordination and cooperation for mutual benefit. It can be understood as the property of an individual (individual social capital), or of a community (ecological social capital) [34–36]. It can also be divided into structural and cognitive social capital [37, 38] or bonding and bridging social capital [38, 39].

#### Confiding relationships

Measures of confiding relationships rate the degree of closeness to and intimacy someone has with another person, or specified other people [40, 41].

#### Alienation

Bronfenbrenner [42] defined alienation as “the feeling of disconnectedness from social settings, such that the individual views his/her relationships from social contexts as no longer tenable”.

### A model of social isolation and related concepts

Our review of conceptual definitions enabled us to generate a conceptual model of social isolation and related terms. Our aim was to develop a set of defined domains that would encompass all the frequently used concepts, avoiding overlap or duplication. We developed and corroborated this model by checking the match of the concepts identified to our model domains, and iteratively consulting experts. We propose five conceptual domains that are comprehensive enough to encompass all elements of current conceptualisations: social network—quantity; social network—structure; social network—quality; appraisal of relationships—emotional; and appraisal of relationships—resources. Table 1 summarizes how these five domains map on to the existing conceptual terms.

**Table 1:** Social isolation and related concepts: conceptual framework

Established concepts relating to social isolation or loneliness	Domains included in the existing concepts relating to social isolation or loneliness					
	Network			Appraisal of relationships		Other domains (not directly related to social isolation or loneliness)
	Quantity	Structure	Quality	Emotional	Resources	
Social isolation	×		×	×	×	
Loneliness				×		
Social support	×	×		×	×	
Social network	×	×	×			
Social capital (individual)				×	×	Ecological social capital, negative social capital
Confiding relationships and related concepts			×			Negative aspects of relationships
Alienation				×		Powerlessness, normlessness

Supplementary Tables 1–7 provide further information about the existing conceptual definitions of social isolation and related terms, and how the components of these definitions map on to our proposed five domains. Definitions of our five conceptual domains are as follows:

*Network (Quantity)* refers to quantity of social contact; e.g., the number of people in someone’s social network, number or frequency of someone’s social contacts over a period of time.

*Network (Structure)* refers to characteristics of social contacts, not involving any appraisal of the quality of the relationship: e.g., network density (how many of the people in someone’s social network also know each other), and the characteristics of someone’s social contacts (e.g., how many are kin, colleagues, mental health staff, or mental health service users).

*Network (Quality)* refers to the perceived quality of relationships. This domain includes measures of the quality of specific important relationships (e.g., partner and parents). It also includes measures of qualitative information about all someone’s individual social contacts (e.g., rating how many of someone’s social contacts are friends, how many could be confided in, and how many would be missed).

*Appraisal of relationships (Emotional)* refers to overall appraisal of the perceived adequacy or impact of relationships: e.g., loneliness or emotional social support. This domain does not directly relate to, and is not measured by, the number of or quality of specific individual relationships.

*Appraisal of relationships (Resources)* refers to perceived overall access to resources from someone’s social relationships: e.g., tangible social support.

Our five domains enable three important distinctions to be made:

i. Objective versus perceived qualities of someone’s social relationships: The ‘Network—size’ and ‘Network—structure’ domains provide quantitative information about the number or structure of social contacts. ‘Network quality’ and the two relationship appraisal domains, by contrast, relate to qualitative appraisal of relationships or social connectedness.
ii. Individual relationships versus overall social/inter-personal connectedness: The three ‘network’ domains in our conceptual map relate to the quantity or quality of individual relationships. Information about these individual relationships may be summed to describe social connectedness and relationships overall. The two ‘appraisal of relationships’ domains relate to subjective evaluation of relationships overall, without direct reference to specific individuals.
iii. Tangible (practical) and intangible (emotional) support from relationships: ‘Appraisal of relationship—emotional’ refers to perceived companionship, love and emotional support derived from social/inter-personal relationships. ‘Appraisal of relationships—resources’ refers to instrumental (or tangible) support obtainable from social/inter-personal relationships.

There are elements of the existing conceptual terms which are not covered by our proposed five conceptual domains. These were excluded as they do not directly relate to social isolation or related concepts:

i. Negative aspects of relationships: social isolation, loneliness, and related concepts are defined by the presence or absence of contact or desired support from relationships, rather than negative aspects of social relationships. However, concepts of relationship quality, including expressed emotion, and some conceptualisations of social capital also consider the actively negative aspects of inter-personal relationships (such as criticism, or overinvolvement), which require the presence of social contact and may occur independently of loneliness (see Supplementary Tables 5 and 6).
ii. Participation in social, economic or political activity: relevant to social inclusion, social exclusion, and included in some conceptualisations of social capital, e.g., most conceptualisations of structural social capital (see Supplementary Table 5).
iii. Degree of trust, perceived shared norms, or beliefs with society or institutions of power: conceptualisations of social capital and alienation both include consideration at societal level of politico-legal and moral norms and requirements and how these are perceived and experienced by individuals (see Supplementary Tables 5 and 7).

**Fig. 1:**
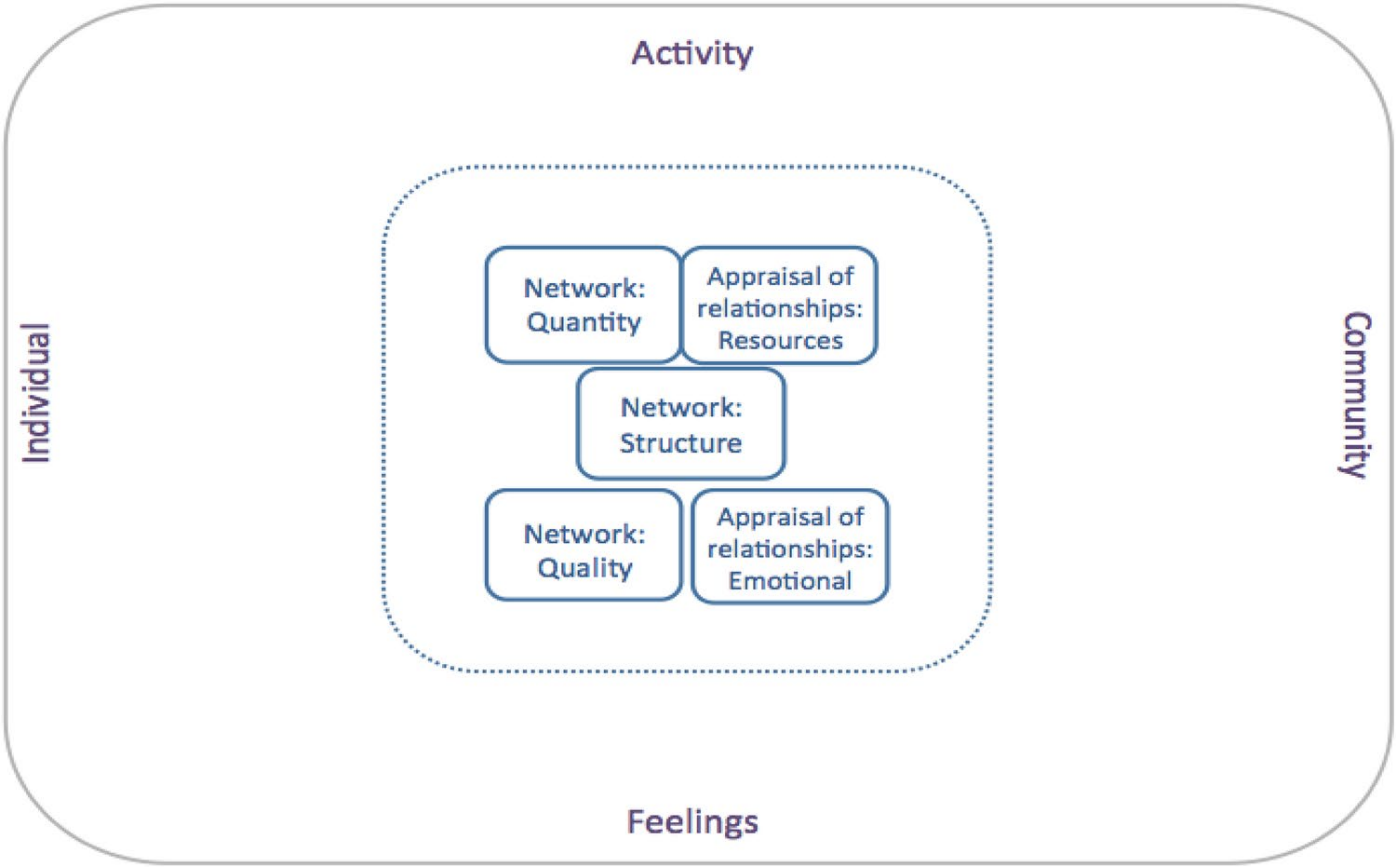
Social isolation and related concepts: conceptual map

Our resulting conceptual map of social isolation and related terms used in mental health research is presented in Fig. 1.

### Measures

First, we describe measures suitable for assessing each of our five proposed conceptual domains of social isolation and related terms (Table 2). Second, we report multi-domain measures used primarily to provide a total score covering more than one of our identified conceptual domains. In both cases, we follow specified criteria in selecting measures, prioritising those which: (1) have been used widely; (2) have adequate psychometric properties; (3) have been used in an adult mental health context.

**Table 2:** Suitable measures of conceptual domains of social isolation and related concepts

Domain	Measure	Description
Network: quantity	Social Network Schedule [43]	*Network size*: the number of people with whom the respondent has had social contact in the last month
		*Frequency of contact*: the number of people whom the respondent has had social contact daily; weekly; or monthly over the past month
Network: structure	Social Network Schedule [43]	*Network density*: the proportion of all possible ties between network members which are present (i.e., how many of a respondent’s network know each other)
		*Proportion of kin*/*non-kin* in social network: How many of the total number of people within a respondent’s social network are relatives?
Network: quality	Social Network Schedule [43]	*Confiding relationships*: the number of social contact people whom the respondent reports they can talk to about worries or feelings
		*Would be missed*: the number of social contacts respondent would miss if never seen again
Appraisal of relationships: emotional	ULS-8 [63]	Eight-item, uni-dimensional scale of *experienced loneliness*
	De Jong-Gierveld Loneliness Scale [64]	11-item scale of experienced loneliness, comprising social and emotional loneliness sub-scales
Appraisal of relationships: resources	Resource Generator-UK [68]	27-item scale assessing a respondent’s access to resources within their social network, comprising four sub-scales: domestic resources, expert advice, personal skills, and problem-solving resources

#### Social network domains

Two measures most widely used to assess social network domains are the Social Network Schedule (SNS) [43] and the Network Analysis Profile (NAP) [44]. The Social Network Schedule (SNS) [43] was designed to assess the social networks of mental health service users. It generates quantitative data for the number of people in someone’s social network; the number of people seen daily, weekly or monthly; the proportion of people in different roles within the network; and the number of people who meet various qualitative criteria, e.g. friends, confidants. The SNS has been used widely and internationally, in both community and inpatient mental health settings [45–50] and has demonstrated good inter-rater reliability [43] and construct validity [51]. Its criterion validity has also been established, with network size and number of confiding relationships associated with quality of life [52], and associated with and predictive of better social functioning [53].

Similar to the SNS, the NAP identifies the attributes of social contacts, the nature of interactions, and characteristics of respondents’ networks. However, the validity and inter-rater reliability of the NAP are less well established than for the SNS, and it is too lengthy for most routine assessment and research contexts [54]. We, therefore, recommend the SNS for assessing all three conceptual domains relating to network properties.

While the SNS is able to measure three of our proposed domains, scores for each measured variable can only be reported separately; no summary total score can be generated. In this way, the SNS is distinct from multi-domain measures described later.

Regarding network quantity, both network size (overall number of contacts seen at least monthly) and frequency of contacts (number of people seen daily/weekly/monthly) are of interest. For network structure, both network density (number of contacts also in contact with each other) and non-kin relationships are of interest as possible indicators of access to “weak ties” [55], which may promote access to information and resources, and recognition of social norms. The number of confidants and social contacts who would be missed has been identified in the SNS as good markers for relationship quality [51], and may be preferable to measuring number of friends, because of the challenges of achieving a consistently understood definition of “a friend” [5, 56].

While the SNS assesses characteristics of all the social contacts in someone’s network, an alternative approach used with the general population [57] and adolescents [58] is to ask respondents to specify and rate the quality of a selected number of their closest relationships. Where such measures can be used to assess any type of relationship, they are potentially useful to provide an aggregate score relating to network quality. Our review did not find measures validated in mental health settings using this approach, but potentially appropriate, well-established relationship quality measures are described in Supplementary File 1, Table 8.

#### Appraisal of relationship domains

Emotional appraisal: Loneliness measures have been well established and used in mental health settings to assess the overall perceived adequacy of relationships in providing emotional support.

The University of California at Los Angeles (UCLA) Loneliness Scale (version 3) [59] is widely used in the general population and clinical research [59–61]. This uni-dimensional, 20-item scale assesses the frequency and intensity of the current experience of loneliness [62]. Good internal consistency and test–retest reliability after 12 months have been established, and good construct validity, comprising convergent and discriminant validity and the validity of a uni-dimensional factor structure [59]. An eight-item short-form version has later been developed, which was also reported as reliable and valid [63].

The de Jong-Gierveld Loneliness Scale is another commonly used loneliness measure. From an original 34-item multidimensional scale, [64], an 11-item scale was developed. This short version is easier to administer and more suitable for lonely and non-lonely respondents [64]. Good psychometric properties have been established [62, 64, 65]. An even shorter six-item version has also been developed for use in large surveys: three items assess emotional loneliness and three assess social loneliness [66, 67].

*Resource appraisal* Instruments exclusively measuring the perceived ability of social contacts to help with access to resources are few. This domain is often included in broader measures of social support or social capital.

The Resource Generator-UK (RG-UK) [68] asks about access to 27 types of informational/practical support, generating a total measure of social network resource access. The scale comprises four sub-scales: domestic resources, expert advice, personal skills, and problem-solving resources. The measure has good validity and reliability [68], and is feasible for use in mental health settings [69]. It is limited by its culturally specific UK context, and is likely to require future adaptation to ensure validity in different eras or cultural contexts [68].

#### Multi-domain measures

Our review also identified numerous measures covering more than one of our proposed conceptual domains. In particular, our review supports Huxley and Webber’s observation [70] that “measures of social support are as varied as the number of investigators”. These measures, while often comprised of sub-scales, generate and typically report a total score. Interpreting scores or the meaning of score change is, therefore, difficult, because measures reflect more than one distinct concept. We describe a number of these multi-domain measures in Supplementary Table 8, prioritising measures most widely used in mental health settings and with demonstrated good psychometric properties.

## Discussion

This review provides an overview of the existing definitions of social isolation and related terms and proposes a conceptual model with five domains to include all elements of current conceptualisations: social network—quantity; social network—structure; social network—quality; appraisal of relationships—emotional; and appraisal of relationships—resources. It identifies measures suitable for assessing each of the five conceptual domains or covering multi-domains.

### Comparison with other conceptual reviews

House and colleagues [23] distinguished two structures of social relationships and support (social integration/isolation and social network structure) and identified three social processes (social support, relational demands and conflicts, and social regulation or control). The domains of “Network: quantity” and “Network: structure” in our conceptual model correspond to the two structure domains in House’s framework, respectively. House’s model is broader in scope than ours as: (1) the negative or conflictive aspects of relationships were not covered in our model; and (2) the regulating or controlling quality of relationships was beyond the scope of our model due to its main focus on societal level rather than individual level relationships. However, House’s model did not include a person’s emotional response to lack of desired social interaction, and thus, loneliness cannot be subsumed under any of its categories. Given the increasing research focus on loneliness, a specific domain—“Appraisal of relationships (Emotional)” to cover this concept is important for future research. In addition, social isolation in House’s model only refers to its external characteristics, while subjective social isolation which refers to personal attitudes not quantifiable by observation was not covered.

Valtorta and colleagues’ conceptual review of social isolation [18] looked at the literature outside the field of mental health, but their findings are highly compatible with ours. The four concepts measured by instruments included in their review (social support, social isolation, social network, and loneliness) were included in our review, which also considered measures of social capital, confiding relationships, and alienation. Valtorta and colleagues propose two domains of social relationships: (1) objective and structural; and (2) subjective and functional. In our model, the domains of “network quantity” and “network structure” describe objective and structural characteristics of social relationships, while “network quality”, and the two “appraisal of relationships” domains in our model describe functional and subjective characteristics.

Compared with the House’s and Valtorta’s conceptual framework, our model offers two further important conceptual distinctions: (1) the characteristics of a person’s individual social relationships versus their relationships and inter-personal connectedness overall. For example, an individual who has a poor relationship with parents or partner could have enough supportive friendships, thereby generally not feeling socially isolated; and (2) emotional versus practical elements of the functional characteristics of social relationships. A more specific and explanatory framework is helpful for mental health research, because it allows distinction between individual difficulties and societal stigma, and it separates the emotional element, where subjective appraisal may be affected by mental illness symptoms, and the practical element that is less likely to be perceived differently because of psychological difficulties. The compatibility of our conceptualisations with the aforementioned two models, despite the different literatures surveyed, provides a degree of validation for all of them across a range of settings. It provides corroboration that suggests that our review was sufficiently thorough and in-depth to develop a robust conceptual model.

### Strengths and limitations

We sought to ensure the validity of our conceptualisation of social isolation and related terms by following an established, iterative process for conducting conceptual reviews [25] and consulting with external experts. Our review provides a model with five domains into which all relevant conceptual terms fit well.

Three limitations relate to the scope of the review. First, we did not include conceptualisations of how people relate to others within the larger social order. Our review synthesised the existing conceptualisations of social isolation and related terms at an individual level rather than looking at their societal context.

Second, conceptualisations or measures which have not been used in mental health settings were outside the scope of our review. Concepts and measures potentially relevant to, but not used in, the field of mental health may, therefore, have been overlooked. The suitability for other population groups of reported measures, which have been used and validated with mental health populations, remains unclear. There are three reasons for focusing on mental health literature in our review: (1) Loneliness and social isolation are of increasing interest in mental health, so there is a large, recent literature to draw on. Our a priori assumption has been that the same concepts have been found useful as in other literature, but we wished to establish that this is the case and investigate whether there are any mental health-specific concepts in use; (2) the literature on social isolation and related concepts is too vast and diffuse to review comprehensively across all fields. Valtorta and colleagues [18] searched relevant literature in the fields of older adults and cardiovascular disease. Our review, therefore, allows a comparison with how the concepts are used in mental health; (3) A secondary aim of our review is to identify appropriate measures for use with mental health population for the concepts that we propose, which are most easily established through a focus on the mental health literature, allowing identification of measures that have proved feasible and acceptable in this population.

Third, social isolation and related terms have been mainly conceptualised as relating to a lack of relationships or positive aspects of the existing relationships. Our review, therefore, did not fully explore how negative aspects of relationships have been defined or measured, and we identified few scales measuring negative characteristics of relationships. When people report “low” social support, their score may reflect either the absence of support from others or the presence of a negative, conflictive relationship [71], but most social support scales are not able to distinguish these potential meanings of low support [72]. An exception is “the Close Persons Questionnaire” [57] which includes items on three types of support—confiding/emotional support, practical support, and negative aspects of support. Portes [35] also proposes the concept of “negative social capital” deriving from peer pressures for exclusive in-group bonding, or high demands from others. Negative aspects of relationships, such as high expressed emotion or inter-personal friction, have been shown to be associated with poor outcomes in schizophrenia and affective disorders [57, 72–75]. The conceptualization and measurement of negative aspects of relationships is a fruitful area for a future review.

Two further potential limitations of the review relate to the search strategy and procedures. First, the initial electronic search was only conducted in Web of Science with time limits 2013–2015, although further relevant studies were identified through review articles and through reading full-text or reference lists of included studies. Before this process was concluded, however, we reached a point where new conceptual definitions of terms or new measures were rarely being identified, suggesting that saturation of novel information had been reached. Second, screening of potentially relevant studies was conducted by a team of researchers, with no formal checks of reliability in researchers’ selection decisions. To mitigate this, study authors (JW and BLE) provided training for all the researchers involved in the literature searching and were consulted in the event of uncertainty about studies’ relevance.

### Implications for research

The boundaries between social isolation and related terms are often blurred, although they can be conceptually categorized within a relatively small number of domains. This is not solely of academic interest: conceptual clarity can support intervention development and evaluation. A range of interventions may be required to address different problems relating to people’s social relationships. Further research is also needed to understand which aspects of people’s social relations are most important in sustaining good mental health or recovering from mental illness. In both cases, precision about what exactly is being studied and how best to measure it is essential.

The need for better evidence regarding the effectiveness of social interventions is widely accepted [76, 77]. Our review can contribute to this in the area of social isolation by helping researchers and intervention developers to specify expected outcomes of interventions and mechanisms of effect more precisely, and measure them appropriately. Conceptual clarity can also help researchers to explore associations between social relationships and other outcomes, and directions of effect, more precisely.

Furthermore, our review identified a gap in the literature on social isolation and related concepts regarding: online social relationships. The concepts and measures of social relationships retrieved for our review rarely included consideration of online social contact. People with mental illness may experience greater social isolation and loneliness compared to the general population [2–4, 11]. However, they appear to use social media and online networking similarly to the general population [78, 79]. It may, therefore, be important to assess online contact when considering social relations in the field of mental health. A systematic review identified limited and primarily qualitative research conducted in this area [80]. In studies measuring online social networking, researchers either designed or adapted the existing questionnaires [81–83], illustrating a lack of validated measure of online social relationships. This lack has hampered comparisons of results across studies [80]: development of such a measure would be a useful focus for future research.

In conclusion, our review proposes a conceptual model with five categories which fits all concepts relevant to social isolation. It can help researchers and practitioners to understand and distinguish these concepts, and how they can best be measured in the field of mental health.

## Electronic supplementary material

Below is the link to the electronic supplementary material.

Supplementary material 1 (DOCX 46 KB)

Supplementary material 2 (DOCX 24 KB)
