# Supplementary material for: Social isolation in mental health: a conceptual and methodological review
Source: Soc Psychiatry Psychiatr Epidemiol. 2017 Oct 28;52(12):1451–61. doi: 10.1007/s00127-017-1446-1 (PMC5702385; doi:10.1007/s00127-017-1446-1)
Supplement: Supplementary file 1 — Supplementary material 1 (DOCX 46 KB) [file 127_2017_1446_MOESM1_ESM.docx]

**Electronic Supplementary Material 1**

**Title:** Social isolation in mental health: a conceptual and methodological review

**Journal:** Social Psychiatry and Psychiatric Epidemiology

**Authors:** Jingyi Wang, Brynmor Lloyd-Evans*, Domenico Giacco, Rebecca Forsyth, Cynthia Nebo, Farhana Mann, Sonia Johnson

* Division of Psychiatry, University College London

Email: b.lloyd-evans@ucl.ac.uk

**Supplementary Tables 1-7: Conceptualisations of social isolation and related concepts in existing literature, and their fit with our proposed domains**

**Supplementary Table 1: Conceptualisations of social isolation**

| **Reference** | **Attributes** | **Fit with proposed domains*** |
| --- | --- | --- |
| Zavaleta et al. (2014) [1] | Internal social isolation  (satisfaction with social relations, need for relatedness, loneliness, feeling of belonging to community; trust) | 4, 5 |
|  | External social isolation  (frequency of social contact; social network support; presence of a discussion partner; reciprocity and volunteering) | 1, 3 |
| Nicolson (2009) [2] | Number of contacts | 1 |
|  | Feeling of belonging | 4 |
|  | Fulfilling relationships | 4 |
|  | Engagement with others | 5 |
|  | Quality of network members | 3 |

* Notes:

1 = Network: quantity 4 = Appraisal of relationships: emotional

2 = Network: structure 5 = Appraisal of relationships: resources

3 = Network: quality 6 = Other domains (not directly related to social isolation or loneliness)

**Supplementary Table 2: Conceptualisations of loneliness**

| **Reference** | **Attributes** | **Fit with proposed domains*** |
| --- | --- | --- |
| Hawkley and Cacioppo (2010) [3]  Peplau and Perlman (1982) [4]  Paloutzian and Ellison (1982) [5] | Perceived deficiencies in quantity of one’s social relationships | 4 |
|  | Perceived deficiencies in quality of one’s social relationships | 4 |
| Weiss (1973) [6] | Social-isolation loneliness  (absence of an engaging social network) | 4 |
|  | Emotional-isolation loneliness  (absence or loss of close attachment relationships) | 4 |
| Kearns et al. (2015) [7] | Feelings  (feeling of being on one’s own associated with not having sufficient intimate and/or other contacts, or contacts of the right type) | 4 |
|  | Circumstances  (an individual’s social contacts and social support both in an everyday sense (who one sees, talks to, etc.) and as a latent resource (knowing who can be relied upon for help or support)) | 5 |
|  | Responses  (a consequence of how people cope with, and respond to, their social situation) | 6 |

* Notes:

1 = Network: quantity 4 = Appraisal of relationships: emotional

2 = Network: structure 5 = Appraisal of relationships: resources

3 = Network: quality 6 = Other domains (not directly related to social isolation or loneliness)

**Supplementary Table 3: Conceptualisations of social support**

| **Reference** | **Attributes** | **Fit with proposed domains*** |
| --- | --- | --- |
| Cohen and Wills (1985) [8] | Structural social support  (existence and form of the social network) | 1, 2 |
|  | Functional social support  (how the network serves to provide different kinds of support) | 4, 5 |
| Barrera et al. (1981) [9] | Tangible forms of assistance  (provision of goods and services) | 5 |
|  | Intangible forms of assistance  (guidance and expressions of esteem) | 4 |
| Barrera (1986) [10] | Social embeddedness  (connections to significant others: measured quantitatively - either by presence or absence of indicators, e.g. married, participating in community groups etc., or through social network analysis) | 1, 2 |
|  | Perceived social support  (self-reported perceived availability and adequacy of supportive ties) | 4 |
|  | Enacted support  (reported receipt of helping activity from others) | 5 |
| Dour et al. (2014) [11]  Wills and Shinar (2000) [12] | Emotional  (a resource who listens and validates) | 4 |
|  | Instrumental  (practical support) | 5 |
|  | Informational  (advice) | 4, 5 |
|  | Companionate  (people with whom to socialise) | 4 |
|  | Feedback  (feedback on community's behavioural expectations) | 4 |
| **Reference** | **Attributes** | **Fit with proposed domains*** |
| (Continued from previous page) | | |
| Dour et al. (2014) [11] | Perceived support | 4 |
|  | Received support  (i.e. how often supportive behaviours are received) | 5 |
|  | Social integration  (diversity/ number of relationships) | 1, 2 |
| House (1981) [13]  Cohen and Hoberman (1983) [14]  Wills (1985) [15] | Emotional support  (caring, love and empathy) | 4 |
|  | Instrumental support  (tangible aid and services) | 5 |
|  | Informational support  (guidance or feedback that can provide a solution to a problem) | 4, 5 |
|  | Appraisal support  (information relevant to self-evaluation) | 4 |
|  | Social companionship  (spending time with others in leisure and recreational activities) | 4 |
| Hand et al. (2014) [16]  Sherbourne and Stewart (1991) [17] | Tangible support  (same as instrumental) | 5 |
|  | Affectionate support  (expressing love and affection) | 4 |
|  | Emotional/ Informational support  (offering empathetic understanding and advice) | 4, 5 |
|  | Positive social interaction support  (having others to do leisure activities with) | 4 |
| Ben-Zur et al. (2014) [18]  Kim et al. (2008) [19] | Emotional assistance  (e.g. sympathy, care) | 4 |
|  | Informative assistance  (e.g. advice) | 4, 5 |
|  | Instrumental assistance  (e.g. financial aid or loans, help with responsibilities) | 5 |
| **Reference** | **Attributes** | **Fit with proposed domains*** |
| (Continued from previous page) | | |
| Melrose et al. (2015) [20]  Haber et al. (2007) [21]  Sarason et al. (1990) [22] | Received support  (quantity of supportive behaviors received by an individual) | 5 |
|  | Perceived support  (both the satisfaction with support and the availability of it) | 4 |
| Lin et al. (2015) [23]  Cutrona and Suhr (1994) [24] | Action-facilitating support  (informational support and tangible aid) | 4, 5 |
|  | Nurturant support  (emotional support and network support) | 4 |
| Yan and Tan (2014) [25]  Berkman et al. (2000) [26]  Wortman and Conway (1985) [27] | Informational support | 4, 5 |
|  | Emotional support | 4 |
|  | Companionship | 4 |
|  | Instrumental assistance | 5 |

* Notes:

1 = Network: quantity 4 = Appraisal of relationships: emotional

2 = Network: structure 5 = Appraisal of relationships: resources

3 = Network: quality 6 = Other domains (not directly related to social isolation or loneliness)

**Supplementary Table 4: Conceptualisations of social network**

| **Reference** | **Attributes** | **Fit with proposed domains*** |
| --- | --- | --- |
| Cohen and Sokolowski (1978) [28] | Morphological characteristics of networks  (quantitative properties of a network: size = number of contacts; degree = average number of links each person in network has with others in the network; density = actual links between network members as a proportion of all possible links) | 1, 2 |
|  | Interactional characteristics of networks  (the nature of relationships: intensity = whether relationships are ‘uniplex’ (one function only) or ‘multiplex’ (more than one function); directionality = who is helping whom in a dyadic relationship) | 3 |
| Burt (1982) [29] | Size | 1 |
|  | Density | 2 |
|  | Boundedness  (the degree to which they are defined by traditional structures like kin, neighbours, work) | 2 |
|  | Homogeneity  (how similar members are to each other) | 2 |

* Notes:

1 = Network: quantity 4 = Appraisal of relationships: emotional

2 = Network: structure 5 = Appraisal of relationships: resources

3 = Network: quality 6 = Other domains (not directly related to social isolation or loneliness)

**Supplementary Table 5: Conceptualisations of** **individual social capital^†^**

| **Reference** | **Attributes** | **Fit with proposed domains*** |
| --- | --- | --- |
| Granovetter (1992) [30]  Putnam (1995) [31] | Structural  (quantity and morphology of social contacts and social participation) | 1, 2, 6 |
|  | Relational  (perceived support, trust and sense of belonging derived from relationships) | 4, 5, 6 |
| Grootaert and Van Bastelaer (2002) [32] | Structural  (established roles, social networks and other structures which can facilitate information sharing and participation) | 1, 2, 6 |
|  | Cognitive  (shared norms, values, trust, attitudes and beliefs) | 4, 5, 6 |
| Nahapiet and Ghoshal (1998) [33] | Structural  (quantity and morphology of social networks) | 1, 2 |
|  | Relational  (perceived support) | 4, 5 |
|  | Cognitive  (shared interpretations or systems of meaning with others (norms)) | 4, 6 |
| Putnam (1996) [34]  Szreter and Woolcock (2004) [35] | Bonding  (“strong ties” with proximal social network, characterized by loyalty, homogeneity and exclusivity) | 4, 5 |
|  | Bridging  (“weak ties” with more distal social network, likely to foster social inclusion and participation) | 5, 6 |
| Bird et al. (2010) [36] | Bonding | 4, 5 |
|  | Bridging | 5, 6 |
|  | Linking  (Relationships/ties to people in formal institutions of power) | 5, 6 |
| Chen et al. (2009) [37] | The extent to which relationships are characterized by:  Durability  Trustworthiness  Resource-rich  Reciprocity | 4, 5 |
| Portes (1998) [38] | Instrumental social capital  (relating specifically to the ability of someone’s relationships and social connections to help them access resources: a sub-component of relational social capital) | 5 |
| **Reference** | **Attributes** | **Fit with proposed domains*** |
| (Continued from previous page) | | |
| Portes (1998) [38] | Negative social capital  (e.g. exclusive in-group bonds such as gang membership may inhibit social contact with others; excessive demands from others in someone’s social network) | 6 |
| Kim and Harris (2013) [39] | Five dimensions of social capital:  Social norms  Trust  Partnership with community  Information sharing  Participation in society | 4, 5, 6 |
| Frank et al. (2014) [40] | Five dimensions of social capital:  Trust  Safety  Cohesion  Engagement  Reciprocity | 4, 5, 6 |

^†^ Social capital may be conceptualized as a characteristic of a community or an individual.

* Notes:

1 = Network: quantity 4 = Appraisal of relationships: emotional

2 = Network: structure 5 = Appraisal of relationships: resources

3 = Network: quality 6 = Other domains (not directly related to social isolation or loneliness)

**Supplementary Table 6: Conceptualisations of** **confiding relationships and related concepts**

| **Reference** | **Attributes** | **Fit with proposed domains*** |
| --- | --- | --- |
| Brown and Harris (1978) [41] | Confiding relationship  (having an intimate partner or other in whom one can confide – i.e. discuss problems and feel listened to) | 3 |
| Langston (1994) [42] | Capitalisation support  (the extent to which a partner or other confidant provides a perceived supportive reaction to a personally meaningful event) | 3 |
| Rook (1987) [43] | Companionship  (presence of companionate relationships within someone’s social network which allow participation in activities, recreational or other, for the purpose of enjoyment (i.e. not about instrumental resources)) | 3 |
| Rusbult et al. (1994) [44] | Relationship quality  (The “Investment model” includes 4 aspects of intimate relationships affecting their quality: overall commitment, satisfaction, quality of alternatives, investment) | 3 |

* Notes:

1 = Network: quantity 4 = Appraisal of relationships: emotional

2 = Network: structure 5 = Appraisal of relationships: resources

3 = Network: quality 6 = Other domains (not directly related to social isolation or loneliness)

**Supplementary Table 7: Conceptualisations of** **alienation**

| **Reference** | **Attributes** | **Fit with proposed domains*** |
| --- | --- | --- |
| Durkheim (1951) [45] | Characteristic of an individual | 4 |
|  | Characteristic of a society | 6 |
| Dean (1961) [46] | Powerlessness  (separation from effective control over his economic destiny; of his helplessness; of his being used for purposes other than his own) | 6 |
|  | Normlessness  (purposelessnes and conflict of norms) | 6 |
|  | Social isolation  (feeling of separation from the group or of isolation from group standards (referring to Durkheim’s concept of ‘anomie’)) | 4, 6 |
| Ifeagwazi et al. (2015) [47]  Seeman (1959, 1975) [48,49]  Moszaros (1970) [50]  Maddi (1967) [51] | Powerlessness  (the expectancy or probability held by the individual that his own behaviour cannot determine the occurrence of the outcomes, or reinforcements, he seeks) | 6 |
|  | Meaninglessness  (the individual is unclear as to what he ought to believe; the individual's minimal standards for clarity in decision-making are not met) | 6 |
|  | Self-Estrangement  (the inability of an individual to find self-rewarding or self-consummatory activities that engage him) | 6 |
|  | Normlessness  (high expectancy that socially unapproved behaviours are required to achieve given goals) | 6 |
|  | Isolation  (assign low reward value to goals or beliefs that are typically highly valued in the given society) | 6 |
| Ifeagwazi et al. (2015) [47]  Ernst and Cacioppo (1999) [52]  Lopez-Calva et al. (2012) [53]  Citrin (1977) [54] | Interpersonal alienation  (feelings of being taken advantage of, being left out of things going on around, people around me would not do much if something happened to me, and feelings that one’s personal thoughts do not matter) | 4 |
|  | Political alienation  (the extent of one’s attachment to the ongoing political order or estrangement from society’s central institutional system of government) | 6 |
|  | Socio-economic alienation  (poverty, limited prospects of sustainable employment, and lack of business opportunities and skills relevant to the market needs) | 6 |

* Notes:

1 = Network: quantity 4 = Appraisal of relationships: emotional

2 = Network: structure 5 = Appraisal of relationships: resources

3 = Network: quality 6 = Other domains (not directly related to social isolation or loneliness)

**Supplementary Table 8: Multi-domain measures relating to social isolation and related concepts**

| **Measure** | **Focus** | **Description** | **Psychometric properties and use** |
| --- | --- | --- | --- |
| Close Persons’ Questionnaire [55] | Social support from close relationships | 14-item. Three subscales: emotional and practical support and negative aspects of relationship. | Moderately good test-retest reliability and some criterion validity (moderate relationship with received social support) established  Participants select and rate their most important close relationships, creating a composite score  Used with general population; not validated for a mental health population |
| Interview Measure of Social Relationships (IMSR) [56] | Personal social resources | Multidimensional: size and density of the primary social network, contacts with acquaintances, adequacy of interaction and supportiveness of relationships, and crisis support. | Good inter-rater reliability, a high degree of temporal stability of close relationships, and good acceptability for use in large-scale surveys of individuals with differing social and educational backgrounds |
| Adapted Social Capital Assessment Tool (A-SCAT) [57]  Short version of the Adapted Social Capital Assessment Tool (SASCAT) [58] | Social capital | 18-item. Two dimensions: structural (‘connectedness’) and cognitive (reciprocity, sharing, trust).  9-item. Two dimensions: structural and cognitive social capital. | ‘Psychometric techniques show SASCAT to be a valid tool reflecting known constructs and displaying postulated links with other variables’; good face and content validity |
| Dean Alienation Scale [46] | Alienation | 24-item. Three subscales: powerlessness, normlessness and social isolation | Strong face validity, construct validity, and acceptable levels of internal consistency reliability established |
| Medical Outcomes Study (MOS) Social Support Scale [17] | Social support | 19-item. Four dimensions: emotional/informational, tangible, affectionate, and positive social interaction. | Reliable (all Alphas >0.91) and fairly stable over time, construct validity hypotheses supported |
| Social Provisions Scale (SPS) [59] | Social support | 24-item. Six dimensions: guidance, reassurance of worth, social integration, attachment, nurturance, and reliable alliance. | A reliable and valid measure with adequate reliabilities and construct validity |
| (Continued from previous page) | | | |
| Interview Schedule for Social Interaction (ISSI) [60] | Social relationships | 52-item. Two dimensions: availability, and adequacy. | Sufficiently valid and reliable, and also sensitive to predictable variations between sociodemographic groups, to justify its use in clinical and epidemiological studies, both in psychiatry and general medicine |
| Abbreviated Duke Social Support Index (DSSI) [61] | Social support | 23-item. Three subscales: social interaction, subjective support, and instrumental support.  11-item. Two subscales: social interaction and subjective support. | High reliability and validity, e.g. high internal consistency and correlated with hopelessness and anxiety |
| Interpersonal Support Evaluation List (ISEL) [14,62] | Social support | 48-item. Four domains: tangible, appraisal, self-esteem, and belonging subscales.  12-item. Three subscales: appraisal, belonging, and tangible social support.  6-item. Two dimensions: emotional and tangible. | Internal consistency and test retest reliability ranging from 0.70-0.80, with moderate intercorrelation |
| Social Supporting Rating Scale (SSRS) [63] | Social Support | 10-item. Three dimensions: objective social support, subjective social support, and utilisation of support | Good reliability and validity |
| Multi-dimensional Scale of Perceived Social Support (MSPSS) [64] | Social support | 12-item. Three subscales: perceived support from family/friends/ significant other. | Internal consistency for the subscales was very high |

**References**

1. Zavaleta D, Samuel K, Mills C (2014) Social Isolation: A conceptual and Measurement Proposal. Working Paper: 67. Oxford Poverty & Human Development Initiative (OPHI), Oxford

2. Nicholson NR, Jr. (2009) Social isolation in older adults: an evolutionary concept analysis. J Adv Nurs 65 (6):1342-1352. doi:10.1111/j.1365-2648.2008.04959.x

3. Hawkley LC, Cacioppo JT (2010) Loneliness matters: a theoretical and empirical review of consequences and mechanisms. Ann Behav Med 40 (2):218-227. doi:10.1007/s12160-010-9210-8

4. Peplau LA, Perlman D (1982) Loneliness: A Sourcebook of Current Theory, Research, and Therapy. Wiley Interscience, New York

5. Paloutzian RF, Ellison CW (1982) Loneliness, spiritual well-being, and quality of life. In: Peplau LA, Perlman D (eds) Loneliness: A sourcebook of current theory, research and therapy. Wiley, New York,

6. Weiss RS (1973) Loneliness: The experience of emotional and social isolation. MIT Press, Cambridge

7. Kearns A, Whitley E, Tannahill C, Ellaway A (2015) Loneliness, social relations and health and well-being in deprived communities. Psychol Health Med 20 (3):332-344. doi:10.1080/13548506.2014.940354

8. Cohen S, Wills TA (1985) Stress, social support, and the buffering hypothesis. Psychol Bull 98 (2):310-357

9. Barrera M, Sandler IN, Ramsay TB (1981) Preliminary Development of a Scale of Social Support - Studies on College-Students. Am J Community Psychol 9 (4):435-447

10. Barrera M (1986) Distinctions between Social Support Concepts, Measures, and Models. Am J Community Psychol 14 (4):413-445

11. Dour HJ, Wiley JF, Roy-Byrne P, Stein MB, Sullivan G, Sherbourne CD, Bystritsky A, Rose RD, Craske MG (2014) Perceived Social Support Mediates Anxiety and Depressive Symptom Changes Following Primary Care Intervention. Depress Anxiety 31 (5):436-442

12. Wills TA, Shinar O (2000) Measuring perceived and received social support. In: Cohen S, Underwood LG, Gottlieb BH (eds) Social Support Measurement and Intervention. Oxford University Press, New York, pp 86–135

13. House JS (1981) Work Stress and Social Support. Addison-Wesley, Reading

14. Cohen S, Hoberman HM (1983) Positive Events and Social Supports as Buffers of Life Change Stress. J Appl Soc Psychol 13 (2):99-125

15. Wills TA (1985) Supportive functions of interpersonal relationships. In: Cohen S, Syme SL (eds) Social support and health. Academic Press, New York, pp 61-82

16. Hand C, Law M, McColl MA, Hanna S, Elliott S (2014) An examination of social support influences on participation for older adults with chronic health conditions. Disabil Rehabil 36 (17):1439-1444. doi:10.3109/09638288.2013.845258

17. Sherbourne CD, Stewart AL (1991) The Mos Social Support Survey. Soc Sci Med 32 (6):705-714

18. Ben-Zur H, Duvdevany I, Issa DS (2014) Ethnicity Moderates the Effects of Resources on Quality of Life for Persons With Mental Illness Living in Community Settings. Psychiatr Rehabil J 37 (4):309-315

19. Kim HS, Sherman DK, Taylor SE (2008) Culture and social support. Am Psychol 63 (6):518-526

20. Melrose KL, Brown GDA, Wood AM (2015) When is received social support related to perceived support and well-being? When it is needed. Pers Individ Dif 77:97-105. doi:10.1016/j.paid.2014.12.047

21. Haber MG, Cohen JL, Lucas T, Baltes BB (2007) The relationship between self-reported received and perceived social support: A meta-analytic review. Am J Community Psychol 39 (1-2):133-144

22. Sarason BR, Sarason IG, Pierce GR (1990) Traditional views of social support and their impact on assessment. In: Sarason BR, Sarason IG, Pierce GR (eds) Social support: An interactional view. Wiley, New York, pp 9-25

23. Lin TC, Hsu JSC, Cheng HL, Chiu CM (2015) Exploring the relationship between receiving and offering online social support: A dual social support model. Inform Manage 52 (3):371-383. doi:10.1016/j.im.2015.01.003

24. Cutrona CE, Suhr JA (1994) Social support communication in the context of marriage: an analysis of couples' supportive interactions. In: Burleson B, Albrecht T, Sarason I (eds) The Communication of Social Support: Messages, Interactions, Relationships, and Community. Sage, Newbury,

25. Yan L, Tan Y (2014) Feeling Blue? Go Online: An Empirical Study of Social Support Among Patients. Inf Syst Res 25 (4):690-709. doi:10.1287/isre.2014.0538

26. Berkman LF, Glass T, Brissette I, Seeman TE (2000) From social integration to health: Durkheim in the new millennium. Soc Sci Med 51 (6):843-857

27. Wortman C, Conway T (1985) The role of social support in adaptation and recovery in physical illness. In: Wortman C, Conway T (eds) Social Support and Health. Academic Press, New York,

28. Cohen CI, Sokolovsky J (1978) Schizophrenia and Social Networks - Ex-Patients in Inner-City. Schizophr Bull 4 (4):546-560

29. Burt RS (1982) Toward a structural theory of action: network models of social structure, perception, and action. Academic Press, New York

30. Granovetter MS (1992) Problems of explanation in economic sociology. In: Nohria N, Eccles R (eds) Networks and organizations: Structure, form and action. Harvard Business School Press, Boston, pp 25-56

31. Putnam RD (1995) Bowling alone: America's declining social capital. J Democr 6 (1):65-78

32. Grootaert C, van Bastelaer T (2002) Understanding and Measuring Social Capital: A Multi-Disciplinary Tool for Practitioners. The World Bank, Washington, DC

33. Nahapiet J, Ghoshal S (1998) Social capital, intellectual capital, and the organizational advantage. Acad Manage Rev 23 (2):242-266

34. Putnam RD (1996) The Strange Disappearance of Civic America. The American Prospect

35. Szreter S, Woolcock M (2004) Health by association? Social capital, social theory, and the political economy of public health. Int J Epidemiol 33 (4):650-667

36. Bird CE, Conrad P, Fremont AM, Timmermans S (2010) Handbook of Medical Sociology. Vanderbilt University Press, Nashville

37. Chen X, Stanton B, Gong J, Fang X, Li X (2009) Personal Social Capital Scale: an instrument for health and behavioral research. Health Educ Res 24 (2):306-317

38. Portes A (1998) Social Capital: Its origins and applications in modern sociology. Annu Rev Sociol 24:1-24

39. Kim BJ, Harris LM (2013) Social Capital and Self-Rated Health Among Older Korean Immigrants. J Appl Gerontol 32 (8):997-1014. doi:10.1177/0733464812448528

40. Frank C, Davis CG, Elgar FJ (2014) Financial strain, social capital, and perceived health during economic recession: a longitudinal survey in rural Canada. Anxiety Stress Coping 27 (4):422-438. doi:10.1080/10615806.2013.864389

41. Brown GW, Harris T (1978) Social Origins of Depression. Tavistock, London

42. Langston CA (1994) Capitalizing on and Coping with Daily-Life Events - Expressive Responses to Positive Events. J Pers Soc Psychol 67 (6):1112-1125

43. Rook KS (1987) Social Support Versus Companionship - Effects on Life Stress, Loneliness, and Evaluations by Others. J Pers Soc Psychol 52 (6):1132-1147

44. Rusbult CE, Drigotas SM, Verette J (1994) The investment model: An interdependence analysis of commitment processes and relationship maintenance phenomena. In: Canary D, Stafford L (eds) Communication and relational maintenance. Academic Press, New York, pp 115-139

45. Durkheim E (1951) Suicide: A Study in Sociology (translated by Spalding, J.). The Free Press, Glencoe, IL.

46. Dean DG (1961) Alienation - its meaning and measurement. Am Sociol Rev 26 (5):753-758. doi:10.2307/2090204

47. Ifeagwazi CM, Chukwuorji JC, Zacchaeus EA (2015) Alienation and Psychological Wellbeing: Moderation by Resilience. Soc Indic Res 120 (2):525-544. doi:10.1007/s11205-014-0602-1

48. Seeman M (1959) On the meaning of alienation. Am Sociol Rev 24 (6):783-791. doi:10.2307/2088565

49. Seeman M (1975) Alienation Studies. Annu Rev Sociol 1:91-123

50. Moszaros I (1970) Marx’s theory of alienation. Merlin Press, London

51. Maddi SR (1967) Existential Neurosis. J Abnorm Psychol 72 (4):311-&

52. Ernst JM, Cacioppo JT (1999) Lonely hearts: Psychological perspectives on loneliness. Appl Prev Psychol 8 (1):1-22. doi:10.1016/s0962-1849(99)80008-0

53. Lopez-Calva LF, Rigolini J, Torche F (2012) Is there such a thing as middle class values? Class differences, values and political orientations in Latin America. CGD Working Paper 286. Center for Global Development, Washington, D.C.

54. Citrin J (1977) Political Alienation as a Social Indicator - Attitudes and Action. Soc Indic Res 4 (4):381-419

55. Stansfeld S, Marmot M (1992) Deriving a Survey Measure of Social Support - the Reliability and Validity of the Close Persons Questionnaire. Soc Sci Med 35 (8):1027-1035

56. Brugha TS, Sturt E, Maccarthy B, Potter J, Wykes T, Bebbington PE (1987) The Interview Measure of Social Relationships - the Description and Evaluation of a Survey Instrument for Assessing Personal Social Resources. Soc Psychiatry 22 (2):123-128

57. Harpham T, Grant E, Thomas E (2002) Measuring social capital within health surveys: key issues. Health Policy Plan 17 (1):106-111

58. De Silva MJ, Harpham T, Tuan T, Bartolini R, Penny ME, Huttly SR (2006) Psychometric and cognitive validation of a social capital measurement tool in Peru and Vietnam. Soc Sci Med 62 (4):941-953. doi:10.1016/j.socscimed.2005.06.050

59. Cutrona CE, Russell DW (1987) The Provisions of Social Relationships and Adaptation to Stress. In: Jones WH, Perlman D (eds) Advances in Personal Relationships, vol 1. JAI Press, Greenwich, CT, pp 37-67

60. Henderson S, Duncan-Jones P, Byrne DG, Scott R (1980) Measuring social relationships. The Interview Schedule for Social Interaction. Psychol Med 10 (4):723-734

61. Koenig HG, Westlund RE, George LK, Hughes DC, Blazer DG, Hybels C (1993) Abbreviating the Duke Social Support Index for Use in Chronically Ill Elderly Individuals. Psychosomatics 34 (1):61-69

62. Cohen S, Mermelstein R, Kamarck T, Hoberman HM (1985) Measuring the functional components of social support. In: Sarason IG, Sarason BR (eds) Social Support: Theory, Research, and Applications. Martinus Nijhoff, Seattle, pp 73-94

63. Cao YP, Zhang YL, Chang DF, Yang SC, Wang GQ (2011) Correlations between self-reported symptoms and psychosocial factors of perpetrators with domestic violence in China: a population-based sample. Chin Med J 124 (4):546-550

64. Zimet GD, Dahlem NW, Zimet SG, Farley GK (1988) The Multidimensional Scale of Perceived Social Support. J Pers Assess 52:30-41
