# Supplementary material for: Social isolation in mental health: a conceptual and methodological review
Source: Soc Psychiatry Psychiatr Epidemiol. 2017 Oct 28;52(12):1451–61. doi: 10.1007/s00127-017-1446-1 (PMC5702385; doi:10.1007/s00127-017-1446-1)
Supplement: Supplementary file 2 — Supplementary material 2 (DOCX 24 KB) [file 127_2017_1446_MOESM2_ESM.docx]

**Electronic Supplementary Material 2**

**Title:** Social isolation in mental health: a conceptual and methodological review

**Journal:** Social Psychiatry and Psychiatric Epidemiology

**Authors:** Jingyi Wang, Brynmor Lloyd-Evans*, Domenico Giacco, Rebecca Forsyth, Cynthia Nebo, Farhana Mann, Sonia Johnson

* Division of Psychiatry, University College London

Email: b.lloyd-evans@ucl.ac.uk

**Appendix 2: Definitions and brief explanation of relevant conceptual terms**

*Social isolation*

Nicholson [1] undertook an evolutionary concept analysis to identify the definition and attributes of social isolation as experienced by older adults. Five aspects of social isolation: were proposed - “number of contacts, feeling of belonging, fulfilling relationships, engagement with others, and quality of network members [1].

Zavaleta and colleagues [2] in a review of social isolation not specific to a mental health context, defined social isolation as “the inadequate quality and quantity of social relations with other people at the different levels where human interaction takes place (individual, group, community and the larger social environment)”. They distinguished two domains of social isolation: external and internal characteristics. External characteristics, also known as objective social isolation, refer to observable social contacts, namely having few or no meaningful relationships with others [3,2]. Conversely, internal characteristics, also labelled as subjective social isolation, refer to personal attitudes not quantifiable by observation, such as trust, satisfaction with relationships and loneliness [2]. The Nicholson and Zavaleta models of social isolation both include objective social contact and subjective perceived adequacy of contact within one overarching construct of social isolation.

Warren [4] proposed four criteria relating to the quality of someone’s social environment and relationships as essential ingredients of social isolation: stigmatised environment (an individual being negatively appraised as different from other people because of appearance, behaviour or tribe), societal indifference, personal-societal disconnection, and personal powerlessness [4].

*Loneliness*

Loneliness can be construed as a painful emotional state that occurs when there is “a discrepancy between…the desired and achieved patterns of social interaction” [5,6,2]. Bekhet and colleagues [7] summarised three common assumptions from various definitions of loneliness: (1) perceived deficiencies in one’s social relationships; (2) a subjective state, different from the objective state of social isolation; and (3) an unpleasant and distressing experience. Loneliness can be regarded as multifaceted.

Another often cited definition of loneliness is a state of negative affectivity accompanying the perception that one’s social needs are not being met by the quantity or especially the quality of one’s social relationships [8,5,9,10].

Weiss [11] also proposed a multidimensional concept of loneliness, categorising loneliness into social or emotional dimensions. Social loneliness derived from inadequate engaging social networks, while emotional loneliness stemmed from the absence of intimate attachment relationships. Based on this categorisation, Weiss [11] conceived a model of loneliness with six components – attachment, social integration, reassurance of worth, reliable alliance, guidance, and opportunity for nurturance. These components were claimed to be necessary in order to avoid loneliness [11].

*Social support*

Two main conceptualisations of social support have been distinguished: functional and structural [12]. The structural perspective emphasises the existence, quantity, and properties of an individual’s social relations [12]. The functional viewpoint attempts to determine which functions are fulfilled by the person’s social relations [12]. The functions most often cited are (1) emotional support which involves caring, love and empathy, (2) instrumental support (referred to by many as tangible support), (3) informational support which consists of information, guidance or feedback that can provide a solution to a problem, (4) appraisal support which involves information relevant to self-evaluation and, (5) social companionship, which involves spending time with others in leisure and recreational activities [13-15]. Many measures of social support assess three components, spanning both structural and functional domains: (a) social network and social integration variables (i.e., diversity/number of relationships), (b) received support (i.e., how often supportive behaviours are received), and (c) perceived support (i.e., support the person believes to be available if he or she should need it) [16,17]. Cobb [18] proposes the mutuality of obligation in relations with others, as well as the functional support received by an individual from others, as a component of social support.

*Social network*

Social network refers to “a specific set of linkages among a defined set of persons, with the additional property that the characteristics of these linkages as a whole may be used to interpret the social behaviour of the persons involved” [19]. Social network analysis can measure "morphological" and "interactional" characteristics of networks [20]. Morphological characteristics refer to quantitative properties of a network. It includes size (number of contacts), degree (average number of links each person in network has with others in the network), and density (actual links between network members as a proportion of all possible links) [20]. Interactional characteristics on the other hand refer to the nature of relationships. They include intensity: whether relationships are "uniplex" (one function only) or "multiplex" (more than one function); and directionality: who is helping whom in a dyadic relationship [20].

*Social capital*

Social capital is generally understood as “a series of resources that individuals earn as a result of their membership in social networks, and the features of those networks that facilitate individual or collective actions” [21-23]. The widely used definition of social capital in health sciences originates with Putnum [23,24]. By analogy with concepts of physical capital and human capital (tools and training that improve individual productivity), social capital refers to “features of social organization such as networks, norms, and social trust that facilitate coordination and cooperation for mutual benefit” [23]. The concept of social capital emphasises multiple dimensions. It can be divided into a behavioural/activity component and a cognitive/perceptual component (cognitive social capital) [24,25]. Five dimensions of social capital have also been proposed: social norms, trust, partnership with the community, information sharing, and political participation [26].

In addition, social capital has both an individual and a collective aspect – a private face and a public face [27]. It can be considered as property of communities (an ecological construct) or of individuals. Individual social capital is most commonly measured by asking individuals about their participation in social relationships (for example, membership of groups) and their perceptions of the quality of those relationships. Two components of social capital have also been proposed [28]: “Bonding” social capital describes closer connections between people with a family connection or shared group identity, and is typically the source of most of someone’s emotional and instrumental social support. “Bridging” social capital describes more distant connections between people not directly linked to friends or family, with distinctions or distance between them – for example people from different classes or ethnic communities. This distinction mirrors that made by Granovetter [29] between “strong ties” and “weak ties” with others in a person’s social network.

*Confiding relationship*

Measures of confiding relationship rate the degree of closeness to and intimacy someone has with other people [30,31]. For example, intimate relationships with a spouse, or with a friend who was seen on a regular basis and could be relied on to give advice, were considered “good confidant”, while “poor or no confidant” refers to conflicted relationships with a spouse, an unsteady relationship or no one to confide in at all [32]. Since their seminal 1978 paper on the social origins of depression, which established the lack of a confiding relationship as a risk factor for depression, Brown and Harris have emphasised the desirability of separating out: the degree of confiding in a relationship, which may be influenced by both parties’ attachment style and perception of the other; and the active emotional support given by a confidant [33]. This mirrors the distinction between perceived and received support in the social support literature.

*Alienation*

Bronfenbrenner [34] defined alienation as “the feeling of disconnectedness from social settings such that the individual views his/her relationships from social contexts as no longer tenable”. Five basic ways where the concept of alienation has been used were discussed by Marxist and existentialist scholars [35-38]: Powerlessness, Meaninglessness, Normlessness, Isolation and Self-Estrangement. Powerlessness originated in the Marxian view that the worker is alienated when the prerogative and means of decision are deprived in capitalist society [35]. In Seeman’s paper, powerlessness can be conceived beyond the industrial sphere as “the expectancy or probability held by the individual that his own behavior cannot determine the occurrence of the outcomes, or reinforcements, he seeks” [35]. Meaninglessness refers to lack of understanding of the events in which an individual is involved, especially “when the individual's minimal standards for clarity in decision-making are not met” [35]. Normlessness is derived from Durkheim's concept of anomie [39]. Seeman [35] defined an anomic situation as one where there is a “high expectancy that socially unapproved behaviors are required to achieve given goals”. Isolation is related to reward values in terms of alienation. Isolated people “assign low reward value to goals or beliefs that are typically highly valued in the given society” [35]. Self-estrangement refers to the inability of an individual to obtain self-rewarding or self-consummatory activities [35].

Dean[40], however, considered alienation as having three main components: Powerlessness, Normlessness and Social Isolation. The last component was conceived as part of Durkheim's conception of anomie - “a feeling of separation from the group or of isolation from group standards” [40]. A 24-item scale was also constructed by Dean to measure these three components [40].

In the study of Ifeagwazi and colleagues [41], emphasis was placed on interpersonal, political and socio-economic domains of perceived alienation. Interpersonal alienation has been associated with social isolation, loneliness and feelings of distrust [42]. The indicators of interpersonal alienation have been reported to include feelings that one’s thoughts does not count, feelings of being left out, being taken advantage of, and receiving no help if something happened [43]. Political alienation and socio-economic alienation refer to the perceived estrangement from major objects in political domain and from socio-economic activities respectively [41]. Among the above domains, interpersonal alienation is of most relevance to our review, with conceptual overlap with definitions of social isolation and social support.

**References**

1. Nicholson NR, Jr. (2009) Social isolation in older adults: an evolutionary concept analysis. J Adv Nurs 65 (6):1342-1352. doi:10.1111/j.1365-2648.2008.04959.x

2. Zavaleta D, Samuel K, Mills C (2014) Social Isolation: A conceptual and Measurement Proposal. Working Paper: 67. Oxford Poverty & Human Development Initiative (OPHI), Oxford

3. de Jong Gierueld J, Tilburg Tu, Dvkstra PA (2006) Loneliness and Social Isolation. In: Vangelisti A, Perlman D (eds) Cambridge handbook of personal relationships. Cambridge University Press, Cambridge, pp 485-500

4. Warren BJ (1993) Explaining social isolation through concept analysis. Arch Psychiatr Nurs 7 (5):270-276. doi:10.1016/0883-9417(93)90004-g

5. Peplau LA, Perlman D (1982) Loneliness: A Sourcebook of Current Theory, Research, and Therapy. Wiley Interscience, New York

6. Goosby BJ, Bellatorre A, Walsemann KM, Cheadle JE (2013) Adolescent Loneliness and Health in Early Adulthood. Sociol Inq 83 (4):505-536. doi:10.1111/soin.12018

7. Bekhet AK, Zauszniewski JA, Nakhla WE (2008) Loneliness: a concept analysis. Nurs Forum 43 (4):207-213

8. Pinquart M, Sorensen S (2001) Influences on loneliness in older adults: A meta-analysis. Basic Appl Soc Psych 23 (4):245-266

9. Hawkley LC, Hughes ME, Waite LJ, Masi CM, Thisted RA, Cacioppo JT (2008) From social structural factors to perceptions of relationship quality and loneliness: the Chicago health, aging, and social relations study. J Gerontol B Psychol Sci Soc Sci 63 (6):S375-384

10. Wheeler L, Reis H, Nezlek J (1983) Loneliness, Social-Interaction, and Sex-Roles. J Pers Soc Psychol 45 (4):943-953

11. Weiss RS (1973) Loneliness: The experience of emotional and social isolation. MIT Press, Cambridge

12. Sanchez Moreno E (2004) Collectivize social support? Elements for reconsidering the social dimension in the study of social support. Span J Psychol 7 (2):124-134

13. House JS (1981) Work Stress and Social Support. Addison-Wesley, Reading

14. Cohen S, Hoberman HM (1983) Positive Events and Social Supports as Buffers of Life Change Stress. J Appl Soc Psychol 13 (2):99-125

15. Wills TA (1985) Supportive functions of interpersonal relationships. In: Cohen S, Syme SL (eds) Social support and health. Academic Press, New York, pp 61-82

16. Dour HJ, Wiley JF, Roy-Byrne P, Stein MB, Sullivan G, Sherbourne CD, Bystritsky A, Rose RD, Craske MG (2014) Perceived Social Support Mediates Anxiety and Depressive Symptom Changes Following Primary Care Intervention. Depress Anxiety 31 (5):436-442

17. Hupcey JE (1998) Clarifying the social support theory-research linkage. J Adv Nurs 27 (6):1231-1241

18. Cobb S (1976) Social Support as a Moderator of Life Stress. Psychosom Med 38 (5):300-314

19. Mitchell JC (1969) The Concept and Use of Social Networks. In: Mitchell JC (ed) Social Network in Urban Situations. Manchester University Press, Manchester, England, pp 1-50

20. Cohen CI, Sokolovsky J (1978) Schizophrenia and Social Networks - Ex-Patients in Inner-City. Schizophr Bull 4 (4):546-560

21. McKenzie K, Whitley R, Weich S (2002) Social capital and mental health. Br J Psychiatry 181:280-283

22. Portes A (1998) Social Capital: Its origins and applications in modern sociology. Annu Rev Sociol 24:1-24

23. Putnam RD (2000) Bowling alone: The collapse and revival of American community. Simon & Schuster, New York

24. De Silva MJ, McKenzie K, Harpham T, Huttly SRA (2005) Social capital and mental illness: a systematic review. J Epidemiol Community Health 59 (8):619-627

25. Bain K, Hicks N Building social capital and reaching out to excluded groups: the challenge of partnerships. In: CELAM meeting on the struggle against poverty towards the turn of the millennium, Washington DC, 1998.

26. Kim BJ, Harris LM (2013) Social Capital and Self-Rated Health Among Older Korean Immigrants. J Appl Gerontol 32 (8):997-1014. doi:10.1177/0733464812448528

27. Putnam RD (1999) Civic Disengagement in Contemporary America. Government and Opposition/Leonard Schapiro lecutre. London School of Economics,

28. Siegler V (2015) Measuring National Well-being - An Analysis of Social Capital in the UK. Office for National Statistics, London

29. Granovetter MS (1973) The Strength of Weak Ties. Am J Sociol 78 (6):1360-1380

30. Brown GW, Harris T (1978) Social Origins of Depression. Tavistock, London

31. Murphy E (1982) Social Origins of Depression in Old-Age. Br J Psychiatry 141 (AUG):135-142

32. Emmerson JP, Burvill PW, Finlayjones R, Hall W (1989) Life Events, Life Difficulties and Confiding Relationships in the Depressed Elderly. Br J Psychiatry 155:787-792

33. Brown GW, Andrews B, Harris T, Adler Z, Bridge L (1986) Social support, self-esteem and depression. Psychol Med 16 (4):813-831

34. Bronfenbrenner U (1979) The ecology of human development: Experiment by nature and design. Harvard University Press, Cambridge, Mass

35. Seeman M (1959) On the meaning of alienation. Am Sociol Rev 24 (6):783-791. doi:10.2307/2088565

36. Seeman M (1975) Alienation Studies. Annu Rev Sociol 1:91-123

37. Moszaros I (1970) Marx’s theory of alienation. Merlin Press, London

38. Maddi SR (1967) Existential Neurosis. J Abnorm Psychol 72 (4):311-&

39. Durkheim E (1997 [1897]) Suicide, trans JA Spaulding. The Free Press, New York

40. Dean DG (1961) Alienation - its meaning and measurement. Am Sociol Rev 26 (5):753-758. doi:10.2307/2090204

41. Ifeagwazi CM, Chukwuorji JC, Zacchaeus EA (2015) Alienation and Psychological Wellbeing: Moderation by Resilience. Soc Indic Res 120 (2):525-544. doi:10.1007/s11205-014-0602-1

42. Ernst JM, Cacioppo JT (1999) Lonely hearts: Psychological perspectives on loneliness. Appl Prev Psychol 8 (1):1-22. doi:10.1016/s0962-1849(99)80008-0

43. Lopez-Calva LF, Rigolini J, Torche F (2012) Is there such a thing as middle class values? Class differences, values and political orientations in Latin America. CGD Working Paper 286. Center for Global Development, Washington, D.C.
